# An in vivo model allowing continuous observation of human vascular formation in the same animal over time

**DOI:** 10.1038/s41598-020-80497-6

**Published:** 2021-01-12

**Authors:** Yohei Tsukada, Fumitaka Muramatsu, Yumiko Hayashi, Chiaki Inagaki, Hang Su, Tomohiro Iba, Hiroyasu Kidoya, Nobuyuki Takakura

**Affiliations:** Department of Signal Transduction, Research Institute for Microbial Diseases, Osaka University, 3-1 Yamada-oka, Suita, Osaka 565-0871 Japan

**Keywords:** Cell biology, Molecular biology, Imaging, Microscopy

## Abstract

Angiogenesis contributes to numerous pathological conditions. Understanding the molecular mechanisms of angiogenesis will offer new therapeutic opportunities. Several experimental in vivo models that better represent the pathological conditions have been generated for this purpose in mice, but it is difficult to translate results from mouse to human blood vessels. To understand human vascular biology and translate findings into human research, we need human blood vessel models to replicate human vascular physiology. Here, we show that human tumor tissue transplantation into a cranial window enables engraftment of human blood vessels in mice. An in vivo imaging technique using two-photon microscopy allows continuous observation of human blood vessels until at least 49 days after tumor transplantation. These human blood vessels make connections with mouse blood vessels as shown by the finding that lectin injected into the mouse tail vein reaches the human blood vessels. Finally, this model revealed that formation and/or maintenance of human blood vessels depends on VEGFR2 signaling. This approach represents a useful tool to study molecular mechanisms of human blood vessel formation and to test effects of drugs that target human blood vessels in vivo to show proof of concept in a preclinical model.

## Introduction

Angiogenesis, the development of new blood vessels from preexisting vasculature, is essential under many physiological and pathological conditions^1^. Dysregulated angiogenesis contributes to numerous malignant, ischemic, inflammatory, infectious and immune diseases^2^. While an understanding of blood vessel formation is needed to treat these diseases, vascular development and maintenance is a complex process that is difficult to recapitulate in vitro. Because of this, in vivo models are needed for the study of vascular change and drug development. Experimental mouse models that better represent pathological conditions have been generated for a improved understanding of angiogenesis and therapy^3,4^. However, it is difficult to translate experimental results from mouse to human blood vessels. For example, a single cell analysis revealed that endothelial cells are genotypically heterogeneous, and human and mouse endothelial cells have different molecular expression patterns^5^. A previous report showed that tumor xenografts vascularized with human blood vessels grow more rapidly and are more resistant to chemotherapeutic or anti-angiogenic drugs than are murine blood vessels^6^. Thus, human blood vessel models are required to understand human vascular biology and to bridge preclinical testing to clinical trials for drug development.

Recently, several in vivo models of human blood vessels have been developed using both human endothelial cells (ECs) and human tissues. These models include transplanting human umbilical vein endothelial cell (HUVECs) spheroids subcutaneously^7^, mesenchymal precursor cells and EC-derived blood vessels^8,9^ or human stem cell-derived vascular organoids^10^. Although human blood vessels were detected in these human tumor tissue transplant models, they are rapidly replaced by murine blood vessels^11,12^. Futhermore, in such transplantation models, detection of human tumor blood vessels has been accomplished on the basis of immunohistochemistry and/or immunofluorescence using sections of harvested tissues. These methods provide a snapshot of a dynamiclly changing system of spatial interactions and molecular landscapes; however, these methods cannot provide images of the kinetics of changes to the vasculature within the tissue of the same individual mouse. To solve this problem, in vivo imaging is a useful method because this technique allows continuous observation of the blood vessels in a non-invasive manner^13^. Recently, Meehan et al*.* developed a human subcutaneous fat transplantation model to visualize vascular cytoadherence of erythrocytes infected with the human parasite *Plasmodium falciparum* over a short period of time^14^. While tissue-derived human blood vessel engraftment has been successful, to the best of our knowledge, continuous observation of human tissue-derived blood vessels for an extended period of time has not yet been reported.

In the current study, to develop a model allowing the continuous observation of human tissue-derived blood vessels, we used a human tumor tissue transplantation approach because tumor blood vessels have highly angiogenic potential. We chose the cranial window model to monitor human blood vessels because this uses skull bone for fixing the cover glass to enable a stable rigid fixation allowing long-term observation for up to a year^15^. Using this method, we were able to observe human blood vessels in tumor tissues over the long term, for at least 49 days; thus, given the lack of cancer cell growth in our model, we were able to investigate human angiogenesis in the absence of tumor cells. Here, we have assessed whether anti-human VEGFR2 antibody affects blood vessel formation in this model and we discuss its suitability for use in drug screening for new modulators of human angiogenesis by allowing observations on three dimensional blood vessel structures over time.

## Methods

### Ethics statement

Informed consent was obtained from patients for the use of their tumor tissues for transplantation. This study was conducted under recognized ethical guidelines (Declaration of Helsinki) and approved by Osaka University Institutional Review board. Human tumor tissues were distributed by a human tissue bank (the JCRB Bioresouce Bank, National Institutes of Biomedical Innovation, Health and Nutrition, Osaka, Japan). The clinical characteristics of the four human tumor tissues are provided in Table 1.

**Table 1:** Clinical characteristics of the tumors.

Patient no	Race	Sex	Age	Primary organ	Location	Histology	Localized/metastatic	Prior treatment
#1	Asian	Female	67	Colon	Sigmoid	Adenocarcinoma (tub1)	Localized	No
#2	Asian	Male	71	Colon	NA	Adenocarcinoma (tub2)	Localized	No
#3	Asian	Male	83	Colon	Sigmoid	Adenocarcinoma (tub2)	Localized	No
#4	Asian	Male	80	Gastric	Gastroesophageal junction	Adenocarcinoma (muc > sig)	Localized	No

*tub1* tubular adenocarcinoma well differentiated type, *tub2* tubular adenocarcinoma moderately differentiated type, *sig* signet-ring cell carcinoma, *muc* mucinous adenocarcinoma, *NA* not available.

### Mice

NOD.CB17-*Prkdc*^*scid*^/Jcl (NOD-Scid) mice were purchased from CLEA Japan (Tokyo, Japan). All animals were housed in a temperature-controlled environment with a 12 h light/dark cycle, and food and water was freely provided. All mouse experiments were performed under the guidelines of the Osaka University Committee for Animal and Recombinant DNA Experiments (Approval number 4062).

### Transplantation of human tumor tissues into the cranial window

**Figure 1:**
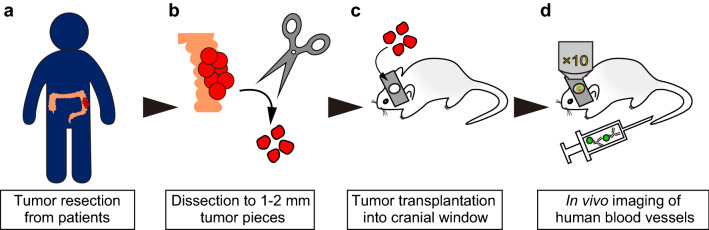
Development of a continuous imaging model of human blood vessels using human tumor tissues. (**a**) Human tumor was freshly resected from cancer patients. (**b**) Tumors were cut into 1–2 mm fragments with scissors. (**c**) Tumor fragments were transplanted into a cranial window in NOD-Scid mice. (**d**) Mice were intravenously injected with Alexa Fluor 488-conjugated anti-human CD31 monoclonal antibody (huCD31-AF488 Ab) to visualize the human blood vessels specifically in vivo. AF488-labeled human blood vessels were observed by confocal microscopy under isoflurane anesthesia.

An overview of the methodology used to generate the xenograft model is shown in Fig. 1. Freshly isolated human tumor tissue was washed twice with HBSS (Thermo Fisher Scientific, Waltham, MA) containing 100 μg/ml kanamycin sulfate (FUJIFILM Wako Pure Chemical Corporation, Osaka, Japan), and 0.5 μg/ml amphotericin B (Thermo Fisher Scientific). It was then was shipped within 2 h after surgery in HBSS containing kanamycin, amphotericin B. On arrival, tumor tissue was washed × 4 with saline (Otsuka Pharmaceutical Factory, Tokushima, Japan) and then cut into 1–2 mm pieces in DMEM (high glucose, no glutamine, no phenol red) (Thermo Fisher Scientific) containing 10% heat-inactivated-FBS (Sigma-Aldrich), 100 units/mL penicillin/100 μg/mL streptomycin (p/s, Sigma-Aldrich). Tumor fragments were placed in HBSS containing 100 ng/mL recombinant human VEGF165 (PeproTech, Rocky Hill, NJ), 100 ng/mL recombinant human bFGF (Peprotech), and p/s. NOD-Scid mice were anesthetized with three mixed anesthetic agents including 0.3 mg/kg of medetomidine (Domitor, Nippon Zenyaku Kogyo, Fukushima, Japan), 4.0 mg/kg of midazolam (Sandoz, Holzkirchen, Germany), and 5.0 mg/kg of butorphanol (Vetorphale, Meiji Seika Pharma, Tokyo, Japan). To prevent cerebral edema during surgery, mice were injected intraperitoneally with 0.2 mg/kg dexamethasone sodium phosphate (Dexart, Fuji Pharma Co., Tokyo, Japan), 5 mg/kg carprofen (Rimadyl, Zoetis, Parsippany-Troy Hills, NJ), 300 μL 10% glycerin and 5% fructose (Glyceol, Chugai Pharmaceutical, Tokyo, Japan). Holes were drilled in the skulls of anesthetized animals to expose the dura mater by slowly lifting the central islands of skull bone. The human tumor fragments were transplanted onto the dura mater. The transplanted areas were filled with artificial cerebrospinal fluid (Artcereb, Otsuka Pharmaceutical Factory) and sealed with a silicon-coated 5 mm round glass cover slip (Matsunami Glass Ind., Osaka, Japan) using a medical adhesive (Aron Alpha A, Daiichi Sankyo, Tokyo, Japan). Mice were kept on a heated pad set to 37 °C until adhesion between glass and skull bone was complete, and then the anesthetic was reversed with 0.3 mg/kg of atipamezole (Antisedan, Nippon Zenyaku Kogyo). To transplant tumor tissues at different time points, samples were preserved in Cellbanker 1plus (Takara Bio, Shiga, Japan) until use.

### In vivo visualization of human blood vessels

In vivo visualization of human blood vessels was performed with a TCS/SP8 (Leica Microsystems, Wetzlar, Germany) equipped with a Chameleon laser (Coherent, Santa Clara, CA). To specifically visualize the human blood vessels, Alexa Fluor 488-conjugated anti-human CD31 monoclonal antibody (huCD31-AF488 Ab, clone: MW59, 303110, Biolegend, San Diego, CA) was injected intravenously. The huCD31-AF488 antibody was allowed to circulate for 7 days and establish stable fluorescence before imaging started. To study in vivo blood flow through the human blood vessels, mice were injected with 1 mg 70 kD Rhodamine B isothiocyanate-dextran (Sigma-Aldrich) dissolved in 100 μL PBS and then observed by microscopy. During the imaging, the animal’s head was immobilized using a custom-made cranial window holder. Mice were kept on a heated pad set to 37 °C, and anesthesia was maintained with isoflurane vaporizer and scavenger (Muromachi Kikai, Tokyo, Japan). To acquire high-resolution images of whole transplanted human blood vessels, these regions were subdivided into multiple (nine to eleven) smaller images. These subdivided regions were imaged individually and then combined via stitching to a larger overview.

### Transparentizing tissues with the dura mater

The tranparentizing solution BABB is a solution of 1 part benzoic acid (FUJIFILM Wako Pure Chemical) plus 2 parts benzyl benzoate (FUJIFILM Wako Pure Chemical). Whole-mount tissue samples were stained with Armenian hamster anti-mouse CD31 mAb (MAB1398Z, Merck Millipore, Darmstadt, Germany, dilution 1/1000), and then stained with Alexa Fluor 647-conjugated anti-Armenian hamster pAb (127-605-160, Jackson ImmunoResearch Laboratories, West Grove, PA, dilution 1/1000). Tissues were treated with 50% methanol/PBS, 100% methanol, 50% BABB/methanol twice for 10 min each and with 100% BABB over night.

### Ramucirumab treatment

Mice with successfully engrafted human vessels were divided into two groups, one for ramucirumab treatment and one for the isotype IgG control. Fourteen days after tumor transplantation, mice were treated with 40 mg/kg ramucirumab (Cyramza, Eli Lilly and Co., Indianapolis, IN) or isotype IgG from human serum (I4506, Sigma-Aldrich) intraperitoneally three times per week.

### Immunofluorescence staining

Tissue fixation and staining of sections or tissues with antibodies was performed using the following method. Excised dura mater was fixed with 4% paraformaldehyde (PFA) in PBS for four hours. It was then embedded in FSC22 Blue (Leica Microsystems) and frozen in liquid nitrogen. Subsequently, 14 μm cryosections were obtained via the Kawamoto film method^16^. An Armenian hamster anti-mouse CD31 mAb (dilution 1/200), rat anti-Ki-67 mAb (14-5698-82, Thermo Fisher Scientific, dilution 1/100), biotin-conjugated mouse anti-human nuclei (HuNu) mAb (MAB1281B, Merck Millipore, 1/400), Cy3 conjugated mouse anti-α-smooth muscle actin (αSMA) mAb (C6198, Sigma-Aldrich, dilution 1/100), rabbit anti-cytokeratin 19 mAb (ab52625, Abcam, Cambridge, UK, dilution 1/100), Alexa Fluor 546-conjugated goat anti-rat IgG pAb (A-11081, Thermo Fisher Scientific, dilution 1/200), Alexa Fluor 546-conjugated goat anti-rabbit IgG pAb (A-11010, Thermo Fisher Scientific, dilution 1/200), Alexa Fluor 647-conjugated streptavidin (S32357, Thermo Fisher Scientific, dilution 1/400), Cy3-conjugated goat anti-Armenian hamster IgG pAb (127-165-160, Jackson ImmunoResearch Laboratories, dilution 1/400), and Alexa Fluor 647-conjugated goat anti-Armenian hamster IgG pAb (127-605-160, Jackson ImmunoResearch Laboratories, dilution 1/400) were used for staining. Cell nuclei were visualized with TO-PRO-3 (T3605, Thermo Fisher Scientific, dilution 1/1000) or Hoechst 33342 (Sigma-Aldrich, 1 μg/mL). To avoid non-specific streptavidin/biotin stain, tissues were blocked using the streptavidin/biotin blocking kit (Vector Laboratories, Burlingame, CA) according to the manufacturer’s protocol.

### Image acquisition and analysis

Immunofluorescence images were obtained with confocal microscopy (TCS/SP8 or TCS/SP5; Leica Microsystems). At least 3 images per sample were acquired. Images were processed using the Leica application suite (Leica Microsystems) and Adobe Photoshop CC software (Adobe, San Jose, CA). To quantify the length of blood vessels, CD31-positive immunofluorescence images were analyzed with the computational analysis tool Angiotool^17^. Each calculated value was plotted graphically.

### Cell culture

MS-1 (mouse pancreatic islet endothelial cell line) was purchased from the ATCC (Manassas, VA) and maintained in DMEM (Sigma-Aldrich, St. Louis, MO) supplemented with 5% heat-inactivated-FBS and p/s. HUVECs (human umbilical vein endothelial cells) were purchased from Kurabo (Osaka, Japan) and maintained in HuMedia-EB2 supplemented with HuMedia-EG (Kurabo).

### Flow cytometric analysis

HUVECs and MS-1 cells were harvested by trypsinization and incubated in 4% FCS PBS containing human Fc receptor blocking solution (Biolegend, dilution 1/1000) or mouse Fc receptor blocking solution (Becton, Dickinson and Co., Franklin Lakes, NJ, dilution 1/1000) for 5 min at room temperature. These cells were incubated with anti-huCD31-AF488 Ab (dilution 1/200) and Alexa Fluor 647-conjugated anti-mouse CD31 Ab (moCD31-AF647 Ab, clone: MEC13.3, 102516, Biolegend, dilution 1/200) in 4% FCS PBS at 4 °C for 30 min. After washing with 4% FCS PBS twice, cells were resuspended in 300 µL 4% FCS PBS containing SYTOX Blue (Thermo Fisher Scientific, dilution 1/1000) and analyzed on a BD LSRFortessa (Becton Dickinson). Data were analyzed using FACS Diva software and FlowJo version 10.6.2 (Becton Dickinson).

### Statistical analysis

Data are shown as mean ± SD. Statistical analysis was conducted by a Welch t test or one-way ANOVA with Dunnett’s multiple comparison test using GraphPad Prism 7 statistical software (GraphPad Software, San Diego, CA). A p-value of < 0.05 was judged as significant.

## Results

### Confirmation of specific recognition of human ECs

**Figure 2:**
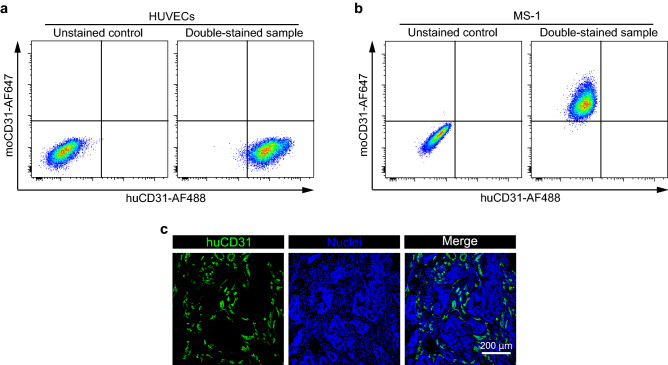
Determination of antibodies that specifically recognize human ECs. (**a**) Specificity of anti-CD31 antibodies for human ECs was analyzed by flow cytometry. Left panel shows unstained HUVECs (negative control) and right panel shows huCD31-AF488 Ab, moCD31-AF647 Ab double-stained HUVECs. (**b**) Specificity of anti-CD31 antibodies for mouse ECs (MS-1 cells) was analyzed by flow cytometry. Left panel shows unstained MS-1, and right panel shows huCD31-AF488 Ab, moCD31-AF647 Ab double-stained MS-1 cells. (**c**) Representative images of colorectal tumor tissues stained with huCD31-AF488 Ab (green). Nuclei (blue) were labeled with TO-PRO-3. Histograms and images are representative of three independent experiments.

To distinguish human from mouse blood vessels in vivo, we tested the commercially available fluorescently-labeled antibody CD31 that recognizes human ECs^14^, and confirmed its species specificity using human ECs HUVECs and mouse ECs MS-1 cells. We tested Alexa Fluor 488-conjugated human CD31 antibody (huCD31-AF488 Ab) and Alexa Fluor 647-conjugated mouse CD31 antibody (moCD31-AF647Ab). Flow cytometric analysis showed that huCD31-AF488 Ab detected the human CD31 extracellular domain and moCD31-AF647 Ab detected the mouse CD31 extracellular domain specifically (Fig. 2a,b). In addition, we confirmed the specificity of the antibody by immunocytochemistry (Supplementary Fig. S1). Next, to determine whether huCD31-AF488 Ab recognizes human blood vessels, we performed immunofluorescence studies on human colorectal tumor tissues. The huCD31-AF488 Ab recognized tumor blood vessels in colorectal tumor (Fig. 2c). Taken together, these results show that huCD31-AF488 Ab allowed the specific observation of human blood vessels.

### In vivo observation of human tumor-derived human blood vessels in the cranial window

**Figure 3:**
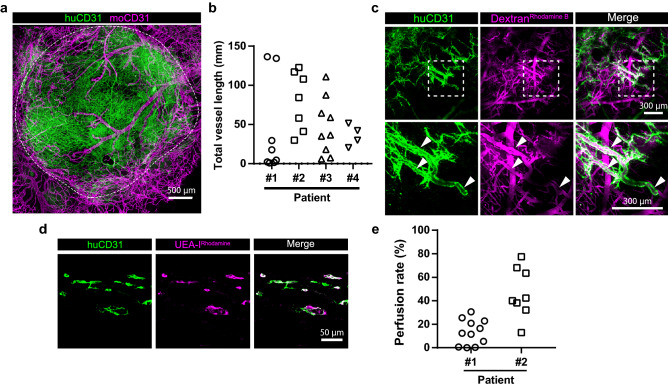
Human tumor-derived human blood vessels in cranial windows. (**a**) Representative image of colon tumor patient-derived human blood vessels (green, huCD31-AF488 Ab injected intravenously) and mouse blood vessels (magenta, anti-moCD31 immunofluorescence staining) in the cranial window. White dashed line shows the edge of the engrafted human tissues on day 19. (**b**) Quantification of total human blood vessel length two weeks after transplantation into cranial the window (Patient #1: n = 8, Patient #2: n = 7, Patient #3: n = 9, Patient #4: n = 4). (**c**) In vivo perfusion of human blood vessels shown by intravenous injection of Rhodamine B-conjugated 70 kDa Dextran (Dextran^Rhodamine B^, magenta) in the tumor xenograft. Image acquisition was at day 20 after tumor transplantation. Lower images show high magnification of the area indicated by dashed boxes in the upper row. White arrowheads show perfused human blood vessels. (**d**) Perfusion of human blood vessels (green, huCD31-AF488 injected intravenously) in the cranial window was assessed by injecting Rhodamine-conjugated *Ulex europaeus* agglutinin I (magenta, UEA-I^Rhodamine^). (**e**) Quantification of perfused human blood vessels in the cranial window (Patient #1: n = 2, Patient #2: n = 2).

Previous reports showed that transplanted human tumor-derived xenografts initially contained human blood vessels^12,18^; however, immunohistochemistry showed that intra-tumor human blood vessels decreased markedly 3 days after transplantation or after in vivo passage to secondary recipient mice. In order to observe the change of human blood vessels in our model, we transplanted human tumors into cranial windows and continuously observed human-specific tumor blood vessels by injecting huCD31-AF488 Ab intravenously. Whole-mount staining showed that huCD31-AF488 Ab allowed visualization specifically of human blood vessels in vivo (Fig. 3a). We transplanted four human tumors (3 colorectal tumors, and one gastric tumor) into cranial windows. After two weeks, in vivo imaging showed that human blood vessels from different tumor patients were engrafted (Fig. 3b, Supplementary Fig. S2a–c). We examined the relationship of engrafted human blood vessels and the original tumor blood vessels (Supplementary Fig. S2d). However, we could not identify any factors that influenced the engraftment of human blood vessels in this model. Next, to determine whether transplanted human blood vessels were functional, we injected Rhodamine B-conjugated 70 kDa Dextran into the tail vein. In vivo imaging showed that human blood vessels were partially perfused (Fig. 3c). To further investigate this, we injected the Rhodamine-conjugated human blood vessel-specific lectin, *Ulex europaeus* agglutinin I (UEA-I^Rhodamine^)^19^. The degree of perfusion through the human blood vessels was different in grafts derived from the different patients (Fig. 3d,e). These results show that tumor-derived human blood vessels survive in a cranial window model and are partially still perfused after 2 weeks.

### Continuous increase of tumor-derived human blood vessels

**Figure 4:**
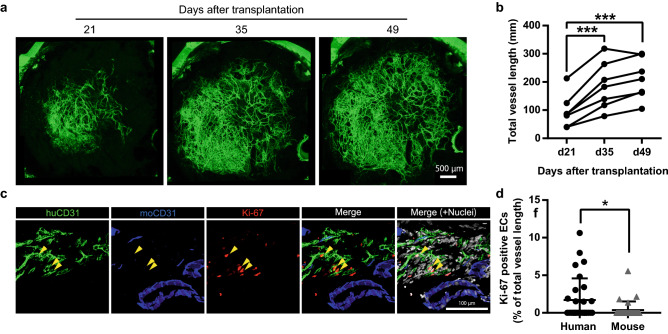
Continuous observation of tumor-derived human blood vessels (**a**) Representative images of continuously expanding human blood vessels derived from patient #1 in the cranial window. (**b**) Quantification of total human blood vessel length in the cranial window. Data for each value were evaluated statistically (n = 7). One-way ANOVA and Dunnett’s multiple comparisons testing was used to compare day 21 and day 35, day 21 and day 49. (**c**,**d**) Ki-67-positive ECs in patient tumor dura mater at day 50 after tumor transplantation. (**c**) Immunofluorescence staining of human ECs (huCD31, green), cell division marker (Ki-67, red), mouse ECs (moCD31, blue). Hoechst staining shows total nuclei (gray). Yellow arrowheads show Ki-67-positive human ECs. (**d**) Quantification of Ki-67-positive human ECs and mouse ECs. Data mean ± SD. (n = 6). Welc’s t test was used to compare with Ki67 positive human ECs between that of mouse. *p < 0.05. ***p < 0.001.

The cranial window model allows continuous observation of blood vessels over time in the same animal in vivo^8^. As seen in Fig. 4a,b, the length of human blood vessels significantly increased from day 21 to day 49, which is inconsistent with results reported in a previous study^11^.In addition, the number of human vessel junctions and average vessel diameter increased (Supplemenrary Fig. S3a,b). Vessel density, on the other hand, was unchanged (Supplemenrary Fig. S3c). Immunofluorescence analysis showed that parts of the human blood vessels are positive for Ki-67 (Fig. 4c). Moreover, Ki-67 positivity was significantly higher in the human than mouse blood vessels (Fig. 4d). These results suggest that proliferation of ECs was induced predominantly in the human ECs in our model. Because transplanted human blood vessels have an immature structure, we determined whether they were covered with α-smooth muscle actin (αSMA)-positive pericytes. The results indicated that the human blood vessels were indeed partially covered with αSMA-positive pericytes (percent of pericyte coverage: 13.8% ± 10.2%) (Supplemenrary Fig. S3d,e). Tumor angiogenesis is facilitated by factors secreted by cancer cells and the surrounding stroma cells, for example vascular endothelial factors (VEGFs) and fibroblast growth factor (FGFs)^20^. However, there were no detectable colorectal cancer cells in this model (Supplemenrary Fig. S3f,g). Thus, these results indicate that tumor-derived human blood vessels have angiogenic potential in the cranial window and that angiogenic signals are derived from non-cancer cells.

### The growth of human blood vessels depends on VEGFR2 signaling

**Figure 5:**
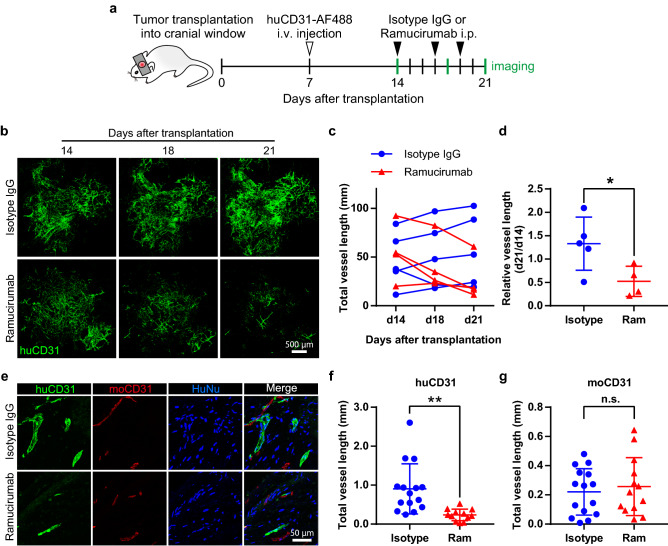
VEGFR2 inhibition causes regression of human blood vessels. (**a**) Schema for tumor transplantation into the cranial window and ramucirumab or isotype IgG treatment. b-d, VEGFR2 inhibition decreases human blood vessels. Representative images of huCD31-AF488-positive human blood vessels (huCD31, green) (**b**). Quantification of total human blood vessel length (**c**) and relative length (**d**) after ramucirumab (Ram) or isotype IgG (Isotype) treatment. (**e**–**g**): Transplanted tissues were sectioned for the analysis of human and mouse blood vessels. Representative images for the expression of human CD31 (huCD31, green), mouse CD31 (moCD31, red), and/or human nuclei (HuNu, blue) (**e**). Quantification of the total length of human (**f**) and mouse (**g**) blood vessels. Data are mean ± SD. (isotype IgG: n = 5, ramucirumab: n = 4). Welch´s t test was used to compare with isotype IgG-treated mice. *: p < 0.05. **: p < 0.01. n.s.: not significant.

Even though several angiogenic factors have been identified, VEGF is one of the most important molecular cues for the formation of new blood vessels^21^. To examine whether growth of tumor-derived human blood vessels in our cranial window model also depends on VEGF signaling pathways, the anti-human VEGFR2 antibody ramucirumab was given three times a week from day 14 to day 21 after tumor transplantation (Fig. 5a). Ramucirumab induced a rapid decrease of human blood vessels (Fig. 5b–d, Supplementary Fig. S4a–c). To determine whether ramucirumab affects only human but not mouse blood vessels, tumor-transplanted dura maters were retrieved on day 21 after transplantation. Ramucirumab treatment decreased human blood vessels but did not inhibit the formation of mouse blood vessels (Fig. 5e–g). It is generally accepted that low-dose anti-angiogenic therapy induces vascular normalization^22^. To test whether ramucirumab does induce vascular normalization, we examined αSMA pericyte coverage. However, we found that ramucirumab did not influence αSMA pericyte coverage in our model (Supplementary Fig. S4d,e). These results show that ramucirumab inhibits VEGFR2 signaling of human blood vessels. Taken together, our data show that human blood vessels in cranial windows depend on VEGFR2 signaling.

## Discussion

To evaluate human blood vessel formation in vivo, most earlier studies compared tumor sections from different mice by immunohistochemistry or immunofluorescence^11,12^. However, these methods only show a snapshot of human blood vessel presence at the time that the tissue was obtained. Here, we developed a method for observing human blood vessels in vivo continuously over time in the same animal. This model of tumor transplantation into a cranial window directly revealed changes to human blood vessels by using in vivo fluorescence imaging techniques.

Previous studies reported no staining with the anti-human CD34 antibody in the colorectal tumor xenograft model on day 30, and that human tumor blood vessels had been replaced by mouse blood vessels^11^. In our current model, human blood vessels remained stable and functional for at least 49 days in vivo. We found that some of the ECs in the tumor had high proliferative potential; thus, given the lack of cancer cell growth in our model, human blood vessels are maintained depending on non-cancer cell-derived VEGF in this cranial window model. Further analysis of the origin of this VEGF is required; however, it is apparent that our method differs significantly from previous reports on the growth of human blood vessels in vivo In a recent study, Koh et al. showed that dura mater ECs had more angiogenic potential than brain ECs, and that VEGFR2 signaling in the dura mater promoted vascular regeneration in an experimental head injury model^23^. Considering these results, damage to the dura mater caused by tumor transplantation may promote VEGF-dependent EC sprouting. We hypothesize an interaction between the mouse dura mater cells and the human ECs for the development of the human vasculature.

We depict the relationship of engrafted human blood vessels with the original tumor blood vessels in Fig. 3b and Supplementary Fig. S2d. We were unable to identify any factors influencing the engraftment of human blood vessels in the present work. Previous reports indicated that pericytes maintain stability of blood vessels via the angiopoietin 1/Tie2 pathway^24^ and induce expression of the anti-apoptotic protein Bcl-w in tumor ECs, thereby protecting them from cytotoxic damage^25^. Therefore, we speculate that engraftment of human blood vessels is dependent on the pericyte coverage of the tumor ECs. In addition, we hypothesize that perfusion of transplanted human blood vessels is dependent on the pericyte coverage of the tumor ECs. To better understand vessel function, it would be necessary to assess vascular permeability, but given the admixture of mouse and human blood vessels in our model, it is difficult to distinguish whether it is the mouse or human blood vessels that are responsible for the permeability. Real-time visualization of vascular permeability is desired and further technical improvement will be required in our model.

We documented perfusion through the human blood vessels in Fig. 3c–e. These results show that the human blood vessels do connect with the murine circulation. Cheng et al*.* showed that HUVEC-derived human blood vessels connect with mouse blood vessels at the host-implant interface and designated this phenomenon “wrapping-and-tapping” (WAT) anastomosis^8^. VE-cadherin is an important transmembrane component at the endothelial cell–cell junction. Therefore, human blood vessels in our model may connect to mouse blood vessels via VE-cadherin. It will be intriguing to clarify how cellular junctions between human and mouse ECs are induced by adhesion molecules and the cytoskeleton.

In terms of experimental benefits, our model requires fewer animals, and provides for noninvasive, continuous evaluation of a drug’s effect over time in the same animal, which conventional methods cannot offer. This model therefore allows the study not only of morphological changes such as the length of the human blood vessels but also functional evaluation, such as the degree of perfusion. It is widely accepted that functional normalization of the tumor vasculature is extremely important for improving drug delivery^26^ and facilitating immune cell access^27^. Our model allows us to examine the perfusion status of the human blood vessels by using a fluorescence-labeled lectin which specifically binds to human vascular ECs. Our current model would be beneficial for examining the morphological changes of functional human blood vessels exposed to drugs that target tumor blood vessels, which improves the translational potential of pre-clinical animal studies.

An interesting characteristic of our model is that cancer cells cannot be engrafted; however, human blood vessels can be maintained. At present, it is difficult why cancer cells are not engrafted, it is possible that our in vivo model may repulse human cancer cells by unknown immune-like cells. As a tool for screening of drugs affecting human vasculature, our model has benefits. On the other hand, when tumor vasculature needs to be observed in combination with the growth of cancer cells, our model would not be appropriate. To solve this problem, we may need several methodological improvements, e.g. shortening the time required for transportation of the tumor sample from the hospital where patients received surgical resection, using high-grade cancer tissues (i.e. those with greater proliferative potential and resistance to immune attack) or using highly immunodeficient mice, especially NOD-Scid interleukin-2 receptor gamma chain null (NOG/NSG) mice instead of NOD-Scid mice^28^. Another approach to enable cancer cell growth in our model may be to transplant cancer organoids^29^ or human mesenchymal stem cells^30^ together with human tissues including human blood vessels.

Even if cancer cells are engrafted in the cranial window, we would not be able to visualize them in this model. Fonnes et al. showed that injection of near-infrared fluorescently-labeled monoclonal antibody targeting epithelial cell adhesion molecule (EpCAM) as in vivo imaging probe (EpCAM-AF680) to patient-derived tumor-bearing mice enabled visualization of tumor^31^. HuCD31-AF488 and EpCAM-AF647 injection may serve as an in vivo visualization of interaction of cancer cells and tumor blood vessels.

In summary, we developed a human blood vessel transplantation model, which should provide a useful tool for studying the molecular mechanisms of human blood vessel formation in vivo and for testing drugs for their effects or side effects on human blood vessels in vivo.

## Supplementary Information

Supplementary Information.

## Data Availability

The datasets generated during and/or analyzed during the current study are available from the corresponding author on reasonable request.
