# Supplementary material for: An in vivo model allowing continuous observation of human vascular formation in the same animal over time
Source: Sci Rep. 2021 Jan 12;11:745. doi: 10.1038/s41598-020-80497-6 (PMC7804448; doi:10.1038/s41598-020-80497-6)

Supplementary Information

An *in vivo* model allowing continuous observation of human vascular formation in the same animal over time

Yohei Tsukada[1], Fumitaka Muramatsu[1], Yumiko Hayashi[1], Chiaki Inagaki[1], Hang Su[1], Tomohiro Iba[1], Hiroyasu Kidoya[1], Nobuyuki Takakura[1,*]

[1]Department of Signal Transduction, Research Institute for Microbial Diseases, Osaka University, 3-1 Yamadaoka, Suita, Osaka 565-0871, Japan
*Correspondence: ntakaku@biken.osaka-u.ac.jp

Methods

Immunocytochemistry

HUVECs and MS-1 cells were grown to confluence and then fixed with 4% PFA and permeabilized using 0.1% Triton X-100. Cells were labeled with mouse anti-huCD31-AF488 mAb (dilution 1/100) and rat anti-moCD31 mAb (dilution 1/100) and visualized with Alexa Fluor 546-conjugated goat anti-rat IgG pAb. Cell nuclei were visualized with TO-PRO-3.

H&E staining

Five µm cryosections were obtained via the Kawamoto film method. Cryosections were stained with Mayer's Hematoxylin Solution (FUJIFILM Wako Pure Chemical Corporation) and 1% Eosin Y Solution (FUJIFILM Wako Pure Chemical Corporation). Subsequently, cryosections were mounted with SCMM-R2 (Section-lab, Hiroshima, Japan).

Figure legends

Supplementary Fig. S1 Immunocytochemistry for determining anti-CD31 species specificities
Specificity of anti-CD31 antibodies analyzed by immunocytochemistry. Representative images of HUVECs and MS-1 cells stained with huCD31 Ab (green) and moCD31 Ab (red). Nuclei (blue) were labeled with TO-PRO-3. Images are representative of three independent experiments.

Supplementary Fig. S2. Quantification of vessel parameters in Fig. 3
(a-c): Quantification of human blood vessel density (a), branching index (b) and average vessel diameter (c) two weeks after transplantation into the cranial window (Patient #1: n=8, Patient #2: n=7, Patient #3: n=9, Patient #4: n=4). (d) Relationships between transplanted human blood vessels and patient tumor blood vessels. Data are mean ± SEM.

Supplementary Fig. S3. Quantification and staining of patient tissue-transplanted dura mater in Fig. 4
(a-c): Quantification of the number of vessel junctions (a), average vessel diameter (b) and vessel density (c) in the cranial window. Data for each value were evaluated statistically (n=7). One-way ANOVA and Dunnett's multiple comparison testing were used to compare day 21 and day 35, day 21 and day 49. (d,e): αSMA-covered ECs in patient tumor dura mater at day 50 after tumor transplantation. (d) Representative images of huCD31 (green), pericyte marker α-smooth muscle actin (αSMA, red), human nuclei (HuNu, blue). (e) Pericyte coverage was calculated as percent of αSMA-positive area per huCD31-positive area. Data are mean ± SD. (f,g): H&E staining (f) and immunofluorescence staining of colorectal cancer cell marker cytokeratin 19 (CK19, red) (g) in the original tumor and corresponding patient-derived tumor dura mater 50 days after transplantation. Images on the right are high magnifications of the area indicated by boxes in the left-hand row. *: $p<0.05$. **: $p<0.01$. ***: $p<0.001$. n.s.: not significant.

Supplementary Fig. S4. Quantification of vessel parameters in Fig. 5
(a-c): Relative values show vessel area (a), number of junctions (b), vessel density (c) after ramucirumab treatment in our model. (d,e): Ramucirumab-treated mouse dura mater tissues were sectioned for the analysis of pericyte coverage. (d) Representative images of huCD31 (green), α-smooth muscle actin (αSMA, red), HuNu (blue). (e) Pericyte coverage was quantified. Data are mean ± SD. (isotype IgG: n=5, ramucirumab: n=4). Welch´s t test was used to compare experimental with isotype IgG-treated mice. *: $p<0.05$. **: $p<0.01$. n.s.: not significant.

Supplementary Fig. S1, Tsukada Y. *et al.*

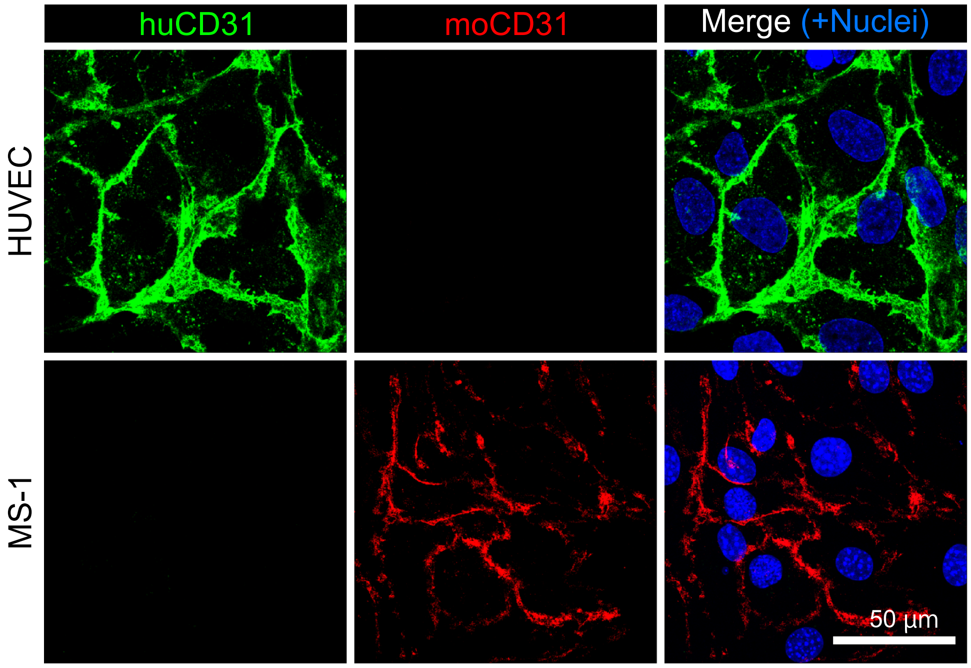

Supplementary Fig. S2, Tsukada Y. *et al.*

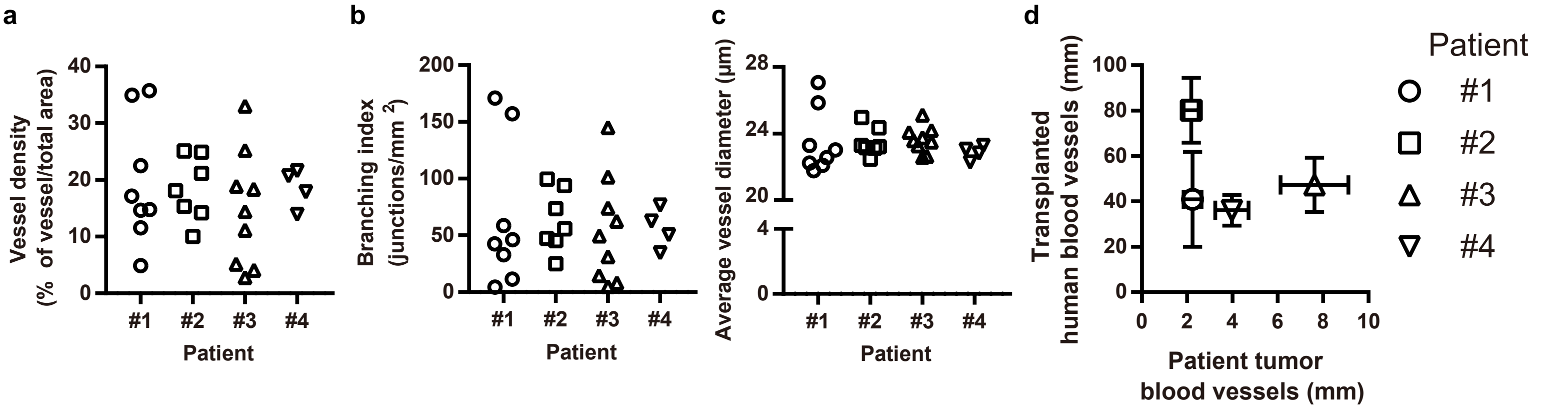

Supplementary Fig. S3, Tsukada Y. *et al.*

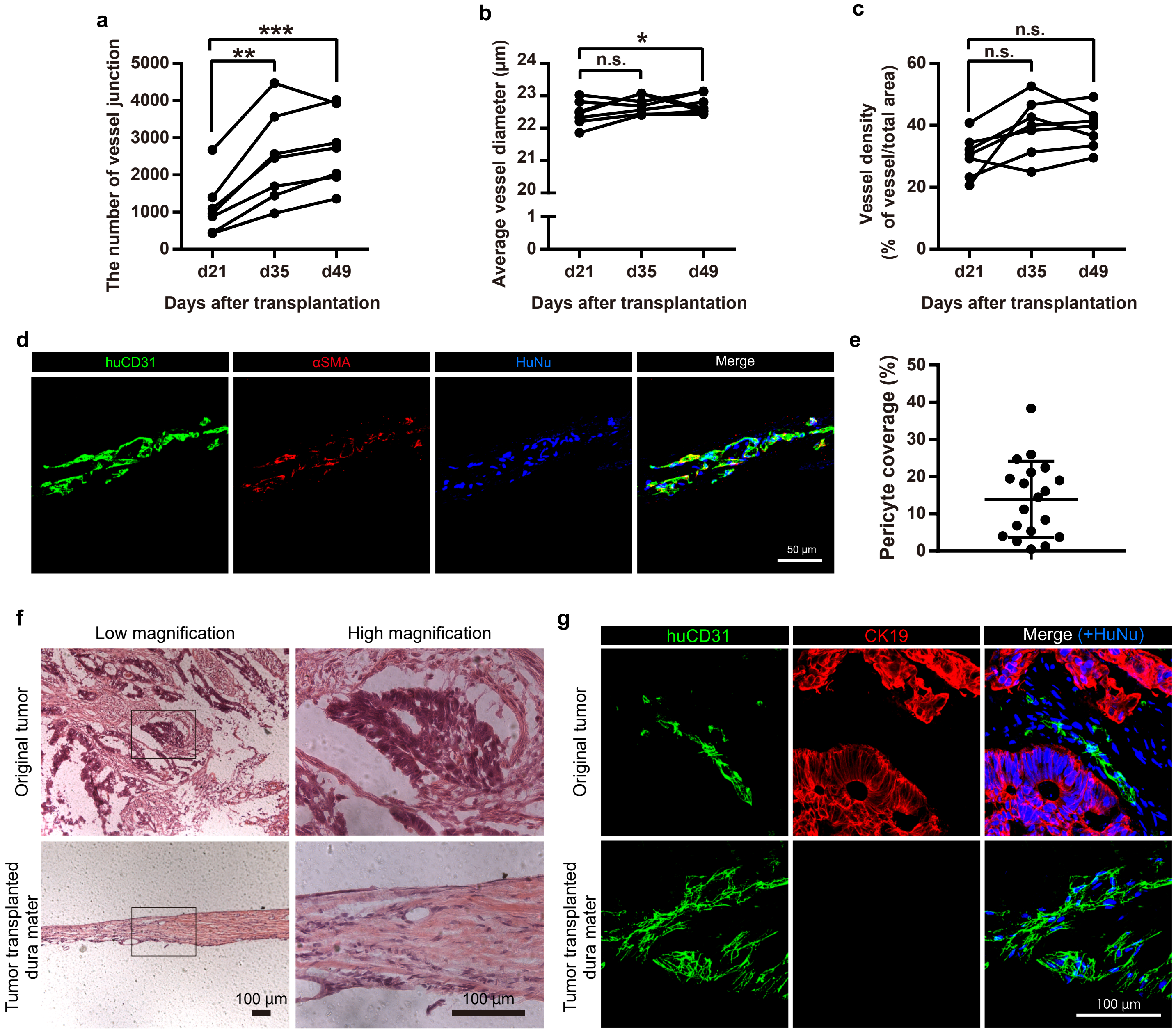

Supplementary Fig. S4, Tsukada Y. *et al.*

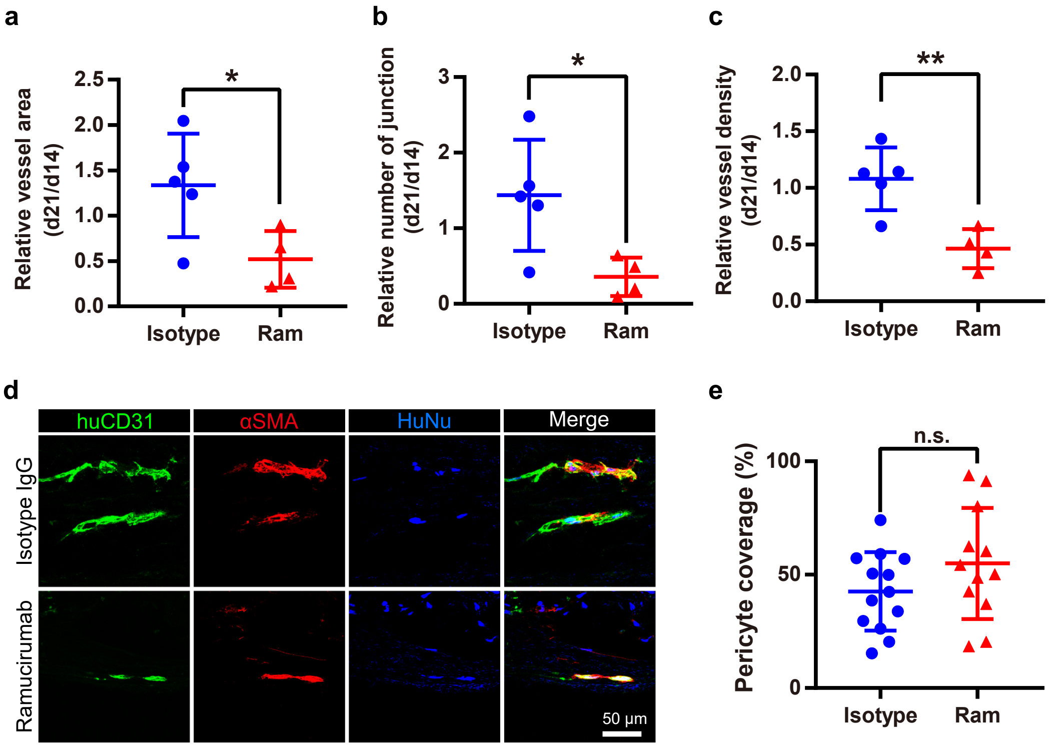

Supplement: Supplementary file 1 — Supplementary Information. [file 41598_2020_80497_MOESM1_ESM.pdf]
